# In Vitro Pharmacokinetic Properties of MK-2048, a Potent Drug Candidate for HIV Prevention

**DOI:** 10.3390/v18050561

**Published:** 2026-05-15

**Authors:** Ruohui Zheng, Guru Raghavendra Valicherla, Phillip W. Graebing, Junmei Zhang, Sharon L. Hillier, Lisa Cencia Rohan

**Affiliations:** 1 Department of Pharmaceutical Sciences, School of Pharmacy, University of Pittsburgh, Pittsburgh, PA 15213, USA; ruz33@pitt.edu (R.Z.); gururaghava810@gmail.com (G.R.V.); pwg12@pitt.edu (P.W.G.); 2 Magee-Womens Research Institute, Pittsburgh, PA 15213, USA; junmei.zhang@pitt.edu (J.Z.); hillsl@mwri.magee.edu (S.L.H.); 3 Department of Obstetrics, Gynecology, and Reproductive Sciences, School of Medicine, University of Pittsburgh, Pittsburgh, PA 15213, USA

**Keywords:** MK-2048, ABC transporters, permeability, drug-metabolizing enzymes, HIV-1

## Abstract

MK-2048 is a potent second-generation HIV integrase inhibitor that has demonstrated acceptable safety and pharmacokinetics (PKs) in clinical trials of vaginal formulations. The substrate-type interactions between MK-2048 and the transporters/metabolizing enzymes that are highly expressed in the human female reproductive tract (FRT) were evaluated. The interactions between MK-2048 and P-gp/BCRP were investigated using a cellular bidirectional permeability assay, while those between MK-2048 and MRP4 were assessed using a vesicular uptake assay. Reaction phenotyping was performed to characterize the interactions between MK-2048 and CYP1A1 and CYP1B1. Using human cervicovaginal fluids (CVFs), MK-2048’s solubility was determined using a thermodynamic solubility method and its protein binding was determined using a rapid equilibrium dialysis method. Our study shows an efflux of MK-2048 in P-gp/BCRP-overexpressing MDCKII cells, which was reduced by a P-gp/BCRP inhibitor. Uptake of MK-2048 in MRP4/control vesicles was found to be ATP-independent. MK-2048 was metabolized by the CYP1A1 enzyme but not by CYP1B1. These data confirm that MK-2048 is a substrate of P-gp, BCRP, and CYP1A1, but is not a substrate of MRP4 or CYP1B1. MK-2048 displays low solubility and high protein binding in human CVF. This data suggests that MK-2048 may potentially interact with drugs that modulate the activity of P-gp, BCRP, and CYP1A1.

## 1. Introduction

In 2024, about 40.8 million people globally were living with HIV and 14% of them were unaware of their infection [1]. A total of 45% of all new HIV infections globally occurred in women and girls, and in high-prevalence areas, such as sub-Saharan Africa, 63% of new HIV infections in 2024 occurred in women and girls (all ages); approximately 3300 adolescent girls and young women aged 15–24 years were newly infected with HIV every week (82.5% of global cases) [1]. Prevention of sexually acquired HIV is essential to reduce new HIV diagnoses and to promote women’s well-being.

Long-acting injectable PrEP using cabotegravir or lenacapavir is highly effective at preventing HIV with superior user adherence [2,3]. However, these injectable agents are costly, in terms of drug procurement and health system support for their delivery, and are not widely available in the areas where HIV infections are most common [4,5]. Daily oral pre-exposure prophylaxis (PrEP) is widely available and can reduce the risk of HIV infection from sexual contact by more than 90% [6]. However, the efficacy of oral PrEP is highly correlated with user adherence [6,7]. The widely used oral tenofovir-based PrEP products, formulated as daily tablets, can produce significant gastrointestinal (GI) discomfort and other side effects, which can potentially lower user adherence [8,9].

The vaginal route for PrEP delivery has been investigated, aiming to provide women with affordable and self-administrated PrEP options [10,11,12]. In several clinical trials, PrEP products administered intra-vaginally, including vaginal gels and rings, demonstrated promising efficacy as well as favorable user acceptance [13,14,15]. In late 2020, a self-administrated vaginal ring containing 25 mg dapivirine (DPV) received a positive opinion from the European Medicines Agency (EMA) for HIV prevention in women as well as WHO prequalification and recommendation as an additional HIV prevention option for women at substantial risk of HIV infection [16]. First approved in Zimbabwe in 2021, the DPV vaginal ring is currently authorized for use in 12 African countries, and the EMA extended its label to include breastfeeding women and adolescent girls aged 16 years and older [17]. This encouraging news has supported the development of additional vaginal PrEP products to provide women with more options for HIV prevention.

MK-2048 is a potent integrase inhibitor that suppresses HIV replication by blocking viral DNA insertion into host DNA. It has been evaluated as a vaginal PrEP candidate and has been shown to be safe in the female reproductive tract (FRT) [18,19]. The first-generation integrase inhibitor raltegravir has demonstrated in vivo efficacy against vaginal HIV challenges [20,21]. However, it has been reported that HIV strains withQ148H/K/R mutations are resistant to raltegravir [22,23]. As a second-generation integrase inhibitor, MK-2048 has shown improved activity against the raltegravir-resistant strains, which makes it a promising drug candidate for HIV prevention [23,24,25]. Nonetheless, in the MTN-027 trial, the cervical tissue MK-2048 concentrations did not translate into HIV protection in an ex vivo challenge assay [18]. Understanding the pharmacokinetic properties of MK-2048, especially the factors affecting its absorption and disposition, are essential to better interpret this clinical finding and inform MK-2048 formulation strategies.

Drug transport, metabolism, and protein binding are important determinants of drug absorption and disposition. Efflux transporters are known to limit drug absorption/penetration and can be responsible for multidrug resistance [26,27,28]. High expression levels of P-gp, BCRP, and MRP4 in the human FRT have been reported and related to in vivo drug–transporter interactions that alter drug distribution [29,30,31,32]. In addition, several integrase inhibitors, including raltegravir, bictegravir, and dolutegravir, have been reported to be substrates for one or more efflux transporters [33,34,35]. We previously reported that nanoparticles containing MK-2048 reduced the efflux ratio (ER) compared to free MK-2048 in MDR1- and BCRP-overexpressing cell lines [36]. However, whether MK-2048 is a substrate of efflux transporters, to be assessed with known transporter inhibitors, remains unknown. Apart from drug transporters, metabolizing enzymes also impact drug disposition and are known to affect the clearance of drugs. Drug-metabolizing enzymes, such as CYP 1A1 and 1B1, have higher expression levels in the human FRT [30,37]. CYP 1A1 and 1B1 were identified as major enzymes for the metabolism of dolutegravir [38]. However, the metabolism of MK-2048 by CYP 1A1 and 1B1 enzymes are still unknown. Moreover, it is well known that protein binding influences the distribution and clearance of drugs. Human cervicovaginal fluid (CVF) contains several proteins, including albumin, alpha-1-acid glycoprotein, mucin glycoproteins, and soluble proteins, which may affect the amount of unbound soluble drug available for tissue absorption [39]. So, it is important to determine the solubility and unbound drug concentration for its pharmacological action in the human FRT. The solubility and protein binding of MK-2048 in human CVF is not known.

The objective of this study was to understand the pharmacokinetic properties of MK-2048 that are responsible for MK-2048 penetration and distribution in the human FRT using in vitro systems. For this purpose, we evaluated the substrate-type interactions of MK-2048 with drug transporters/drug-metabolizing enzymes, its solubility, and its protein binding in human CVF. To our knowledge, this is the first study that formally investigates the interactions of MK-2048 with efflux transporters and metabolizing enzymes, especially those that are highly expressed in the human FRT, following regulatory guidance. A version of this work was previously described in the dissertation thesis of Dr. Ruohui Zheng to fulfill part of her PhD graduation requirements [40].

## 2. Materials and Methods

### 2.1. Materials

MK-2048 and deuterated MK-2048 (*d*_6_-MK-2048) with >99% purity were kindly provided by Merck & Co. (Kenilworth, NJ, USA). Acetonitrile (LC-MS grade) and methanol (HPLC grade) were obtained from Honeywell (Charlotte, NC, USA). Formic acid (LC-MS grade) was obtained from Fisher Scientific (Hampton, NH, USA). Total of 50 mg/mL geneticin (G418 sulfate), fetal bovine serum (FBS), 100× penicillin–streptomycin–glutamine (pen-strep), SuperScript IV First-Strand Synthesis system, Pierce BCA protein assay kit, P-gp (C219) antibody, β-actin (15G5A11/E2) antibody, rabbit anti-mouse IgG secondary HRP antibody, Hank’s balanced salt solution (HBSS), dimethyl sulfoxide (DMSO), human MRP4 vesicles, control vesicles for ABC transporters, rapid equilibrium dialysis device (RED) inserts and MRPs-BCRP vesicular transport assay reagent set were obtained from Thermo Fisher Scientific (Waltham, MA, USA). Dulbecco’s modification of Eagle’s medium (DMEM), 0.25% trypsin and 0.1% EDTA in HBSS (trypsin–EDTA), Dulbecco’s phosphate-buffered saline (DPBS) and recombinant CYP1B1 enzyme were obtained from Corning Inc. (now Discovery Life Sciences LLC) (Corning, NY, USA). Recombinant CYP1A1 enzyme was purchased from Sekisui XenoTech LLC (now BioIVT LLC) (Kansas City, KS, USA). GF120918, Ko143, estradiol 17 β-D-glucuronide (E_2_17βG), β-Nicotinamide-adenine dinucleotide phosphate (NADPH), customized primer oligos, phenacetin, magnesium chloride, orphenadrine, dibasic potassium phosphate, and monobasic potassium phosphate were obtained from Sigma-Aldrich (St. Louis, MO, USA). RNeasy Mini Kit was obtained from Qiagen (Germantown, MD, USA). The 2× Ssofast Evagreen Supermix, 10× Tris/Glycine/SDS buffer, Tween 20, Clarity Max Western ECL substrate, and nonfat dry milk were obtained from Bio-Rad (Hercules, CA, USA). ABCG2 (BXP-21) antibody was obtained from Santa Cruz Biotechnology, Inc. (Dallas, TX, USA). Ultima gold scintillation cocktail and ^3^H-E_2_17βG (45.7 Ci/mmol) were obtained from PerkinElmer (Waltham, MA, USA). MK-571 was obtained from Selleck Chemicals LLC (Houston, TX, USA). 7-Ethoxyresorufin was procured from Cayman Chemical Company (Ann Arbor, MI, USA).

### 2.2. Analysis of Liquid Chromatography with Tandem Mass Spectrometry (LC-MS/MS)

**Figure 1:**
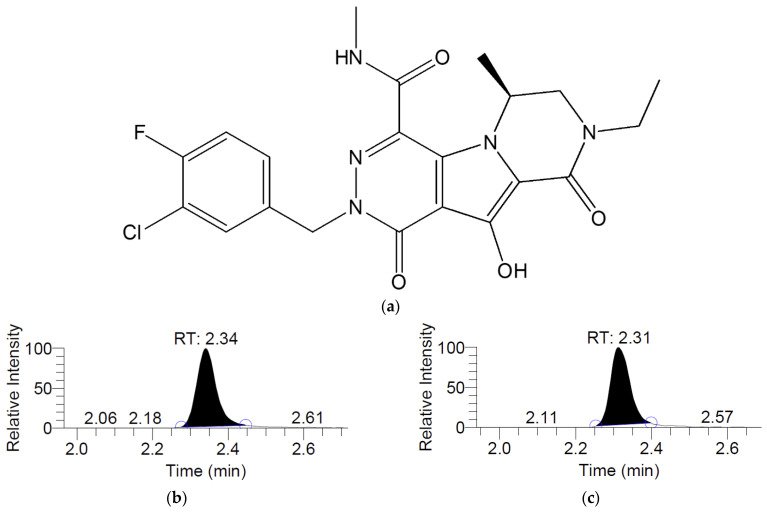
Chemical structure of MK-2048 (**a**), LC-MS/MS chromatograms of MK-2048 (**b**), and internal standard (IS) *d*_6_-MK-2048 (**c**).

The MK-2048 was quantified by LC-MS/MS on Acquity UPLC and Xevo TQ-S MS/MS instruments (Waters Corporation, Milford, MA, USA). A C18 column (Waters Acquity C18 BEH UPLC column, 1.7 μm, 2.3 × 50 mm) was used to achieve chromatographic separation. MassLynx 4.1 software was used to control the LC-MS/MS system and process the acquired data. The mobile phase consisted of 0.1% formic acid in MilliQ water (mobile phase A, 60%) and 0.1% formic acid in acetonitrile (mobile phase B, 40%). The flow rate was set to 0.4 mL/min. The run time for each injection was 4 min. Deuterated MK-2048 (100 nM) was used as the internal standard (IS). Multiple reaction monitoring (MRM) of MK-2048 (*m*/*z* 462→143) and deuterated MK-2048 (*m*/*z* 468→143) were used for quantitation in the positive ion mode (ESI+) with a spray voltage of 3000 V and a capillary temperature under 250 °C. The collected samples were diluted 1:1 with 50% acetonitrile in MilliQ water and injected with a volume of 5 μL. The structure of MK-2048 and the representative chromatograms of MK-2048 and IS are shown in Figure 1. A representative calibration curve is shown in Figure S1. The assay linearity, accuracy, and precision are shown in Tables S1–S3.

### 2.3. Cell Culture

The MDCKII cell lines used in this study were kindly provided by Dr. Philip E. Empey (School of Pharmacy, University of Pittsburgh, Pittsburgh, PA, USA). The wild-type and MDR1-overexpressing MDCKII cells (MDCKII WT and MDCKII MDR1) were cultured in DMEM supplemented with 10% FBS and 1× pen-strep. The BCRP-overexpressing and empty vector-containing MDCKII cells (MDCKII BCRP and MDCKII WT/EV) were cultured in DMEM supplemented with 10% FBS, 800 μg/mL G418 sulfate, and 1× pen-strep. All cell lines were cultured at 37 °C with 5% CO_2_. The cells were cultured until they reached 80% confluence and were then harvested by trypsinization.

### 2.4. qPCR Analysis

The total RNA from the MDCKII cells was extracted with an RNeasy Mini Kit. The cDNA samples were made by reverse transcription with a SuperScript IV First-Strand Synthesis system on an MJ Mini thermal cycler (Bio-Rad, Hercules, CA, USA). The primer sequences were previously reported by Zhou et al. [31]. The expression levels were quantified via qPCR on a CFX96 real-time PCR detection system (Bio-Rad, Hercules, CA, USA). Each 10 μL qPCR reaction mixture contained 10 ng/μL cDNA template, 0.4 μM forward and reverse primers, and 1× Ssofast EvaGreen Supermix. The qPCR was performed with an initial denaturation of 30 s at 95 °C, followed by 40 cycles of denaturation at 95 °C for 5 s and annealing/extension at 60 °C for 30 s. The relative expression levels were calculated using the 2^−ΔΔCq^ method in Bio-Rad CFX Manager software version 3.0, with GAPDH as the reference gene.

### 2.5. Western Blot Analysis

The protein from cell lysates of MDCKII cells was quantified with a Pierce BCA protein assay kit on a SpectraMax M3 plate reader (Molecular Devices, San Jose, CA, USA) and diluted to 3 mg/mL. The diluted protein was fractionated on a Mini-PROTEAN precast gel (Bio-Rad, Hercules, CA, USA) at 100 V for 30 min followed by 150 V for another 30 min. The fractionated protein was then transferred to a nitrocellulose membrane at 100 V for 1 h. After 1 h blocking in PBS with 3% nonfat dry milk, the membrane was incubated overnight at 4 °C with primary antibodies (1:200 for anti-P-gp and anti-BCRP; 1:10,000 for anti-β-actin). On the next day, the membrane was washed and incubated with the secondary antibody (1:10,000) for 1 h at room temperature. The protein was then detected with Clarity Max Western ECL substrate on a ChemiDoc Imager (Bio-Rad, Hercules, CA, USA).

### 2.6. MK-2048 Bidirectional Transport Across MDCKII Cell Monolayers

The MDCKII cells were seeded at a density of 9000 cells per cm^2^ onto 0.4 μm permeable transwell inserts (Corning Inc., Corning, NY, USA). The cells were cultured and allowed to differentiate for 3 to 6 days. The cell growth media was changed every other day and the day before the transport assay. The quality of cell monolayers grown on the permeable inserts was evaluated by measuring the transepithelial electrical resistance (TEER) using a Millicell-ERS apparatus (Millipore, Bedford, MA, USA). Only the monolayers displaying TEER values above the thresholds indicating confluence (380 Ωcm^2^ for MDCKII MDR1 cells and 175 Ωcm^2^ for other MDCKII cells based on the literature-reported values) were used in this study [41,42,43].

To investigate if MK-2048 is a substrate of P-gp and BCRP, bidirectional transport assays, including apical-to-basolateral (A-to-B) transport and basolateral-to-apical (B-to-A) transport, were performed with the qualified MDCKII monolayers. The MDCKII MDR1 and WT cells were used to perform the P-gp study, while the MDCKII BCRP and WT/EV cells were used to perform the BCRP study. The buffer used for the transport assays was HBSS. MK-2048 was investigated at 1.5 μM, a concentration that provided detectable A-to-B flux without the plateauing of B-to-A flux due to the saturated receptor chamber concentration, with and without transporter inhibitors (5 μM GF120918 to inhibit P-gp and 1 μM Ko143 to inhibit BCRP). All the drugs were first dissolved in DMSO and then diluted with HBSS. The final DMSO concentration in each solution was less than 0.1%.

Before each experiment, the monolayers were washed with HBSS. The inhibitor solution or blank HBSS was then added to the donor side for the treated groups or control groups, while the receptor side was incubated with blank HBSS. The volume of the apical chamber was 0.5 mL and that of the basolateral chamber was 1.5 mL. After a 30 min incubation at 37 °C, the MK-2048 solution, with or without a respective transporter inhibitor, was added to the donor side to initiate the transport assay. The cells were incubated with MK-2048 for 90 min in a shaking incubator at 37 °C and 60 rpm. Samples from the donor side were collected at 0 and 90 min. Samples from the receptor side were collected every 15 min for up to 90 min and the same volume of blank HBSS was replenished after each sampling. The collected MK-2048 samples were analyzed with LC-MS/MS as described in Section 2.2.

The apparent permeability coefficient (P_app_) of MK-2048 was calculated using Equation (1), as

(1) $$P_{app}=\frac{dQ/dt}{A\times C_{0}}\;$$

where dQ/dt is the initial linear flux of MK-2048, A is the surface area (1.12 cm^2^) of exposure, and C_0_ is the initial MK-2048 concentration at the donor side. The ER was calculated using Equation (2).

(2) $$ER=\frac{P_{app}\;B\;to\;A}{P_{app}\;A\;to\;B}$$

with the ER values, the net flux ratio was calculated using Equation (3).

(3) $$Net\;flux\;ratio=\frac{ER\;of\;overexpressing\;cell\;line}{ER\;of\;control\;cell\;line}$$

### 2.7. MK-2048 Uptake in Membrane Vesicles

To investigate if MK-2048 is a substrate of MRP4, the ATP-dependent and -independent uptake of MK-2048 was measured with 50 μg MRP4 and control vesicles. A known MRP4 substrate, E_2_17βG, was used as the positive control and MK-571 was used to inhibit MRP4 activity. The uptake assay was performed at a total volume of 50 μL, with either 4 mM ATP or AMP, 2 mM glutathione, and 10 μM MK-2048 or 10 μM E_2_17βG, with and without 20 μM MK-571. For the positive control, 4 μCi/mL ^3^H-E_2_17βG was added for detection. The reaction was initiated by adding the respective assay mixture to the vesicles. The vesicles were then incubated at 37 °C for 3 min. To terminate the reaction, 200 μL ice-cold buffer was added to each well. The samples were then transferred to a glass fiber filter plate and washed 5 times by rapid filtration using a vacuum manifold (IBI Scientific, Dubuque, IA, USA). To quantify the E_2_17βG uptake, the washed vesicles were lysed with scintillation cocktail at room temperature for 20 min. The collected samples were analyzed on a liquid scintillation counter (Beckman Coulter, Brea, CA, USA). To quantify the MK-2048 uptake, the washed vesicles were lysed with 80% methanol in MilliQ water at room temperature for 20 min. The collected samples were analyzed with LC-MS/MS as described in Section 2.2.

If ATP-dependent uptake was observed, the uptake amount by active transport was calculated using Equation (4) as

(4) $$Normalized\;uptake=\left(uptake\;with\;ATP\right)-(uptake\;with\;AMP)$$

### 2.8. MK-2048 Metabolism in rCYP1A1 and rCYP1B1 Enzymes

To investigate if MK-2048 is a substrate of CYP 1A1 and 1B1 enzymes, reaction phenotyping studies were performed with recombinant enzymes as previously described [44]. These experiments were performed in duplicates using silanized glass tubes (Thermo Scientific, Waltham, MA, USA). Recombinant CYP isoforms 1A1 (100 pmol/mL P450) and 1B1 (10 pmol/mL P450) were used as enzyme sources for the reaction incubation. The reaction incubation mixture consisted of an enzyme, 1 mM NADPH, 3.3 mM magnesium chloride, 10 µM MK-2048, and 100 mM phosphate buffer (pH 7.4) in a final volume of 200 µL. The final concentration of DMSO in the reaction mixtures was 0.1%. The reaction was started by the addition of NADPH and incubated for 60 min at 37 °C in a shaking water bath (Thermo Scientific, Waltham, MA, USA). After incubation, the reactions were quenched with 200 µL of acetonitrile containing IS *d*_6_-MK-2048. The samples were vortexed at 1000 rpm for 5 min and then transferred to low-retention microcentrifuge tubes (Fisher Scientific, Waltham, MA, USA). The samples were centrifuged at 15,000 rpm for 15 min to pellet the precipitated protein. The 200 µL supernatant was diluted with 200 µL water and used for the LC-MS/MS analysis. The reaction performed without NADPH (60 min only) was used as a negative control. In this study, phenacetin (10 µM) and 7-ethoxyresorufin (1 µM), respectively known substrates of CYP1A1 and CYP1B1, were used as positive controls. The LC-MS/MS analysis of these two known substrates was performed as described previously [44].

The depletion rate constant (K) was calculated from the % remaining of MK-2048 against time points. The slope of the linear regression equation gives the depletion rate constant. The in vitro half-life (t_1/2_) was calculated as 0.693/K. The in vitro intrinsic clearance (in vitro CL_int_) was calculated using Equation (5).

(5) $$In\;vitro\;{CL}_{int}=\frac{0.693}{t_{1/2}}\times \frac{Volume\;of\;reaction\;mixture\;(mL)}{pmol\;of\;protein}\;$$

### 2.9. Collection of Human Cervicovaginal Fluid

Following the Institutional Review Board approval (IRB: PRO10080337; approval date: 25 June 2019) by the University of Pittsburgh, informed consent was obtained from healthy, asymptomatic, HIV-negative women who were either between 18 and 46 years of age or over the age of 50. A blank informed consent form can be found in Supplemental Information S1. An Instead^TM^ catamenial cup was inserted into the vagina up to the cervix by the clinician and left in place for 6 h. The catamenial cup was removed and placed into a 50 mL conical tube for transport to the laboratory. The collected human CVF was used for solubility and protein binding studies of MK-2048.

### 2.10. Solubility of MK-2048 in Human Cervicovaginal Fluid

The thermodynamic solubility of MK-2048 was determined by adding an excess amount of drug (~5 mg) to 300 µL of human CVF in low-retention microcentrifuge tubes. The solubility study was performed in quadruplet. The tubes were shaken on an orbital shaker at a speed of 500 rpm at 37 °C for 18 h. After incubation, the fluid was centrifuged at 15,000 rpm for 10 min to pellet the excess drug and the supernatant was collected. A total of 50 µL of the collected supernatant, 50 µL of phosphate buffer saline (PBS, pH 7.4), and 100 µL of acetonitrile: methanol (1:1) containing IS *d*_6_-MK-2048 were combined and vortexed for 5 min at 1000 rpm. The samples were centrifuged at 23,100× *g* for 10 min to pellet the precipitated proteins. The 100 µL supernatant was diluted with 500 µL 0.1% formic acid and used for the LC-MS/MS analysis.

### 2.11. Protein Binding of MK-2048 in Human Cervicovaginal Fluid

The protein binding of MK-2048 was determined in human CVF with RED inserts (molecular weight cutoff 8000) in quadruplets using a reported method [45]. A 10 uM final concentration of MK-2048 was prepared in human CVF and the final DMSO concentration was 0.1%. The RED device was filled with 200 µL human CVF with MK-2048 (donor side) and 400 µL PBS (acceptor side). The RED device was sealed and incubated at 37 °C on an orbital shaker at 250 rpm for 6 h. After incubation, the samples were removed from the donor and acceptor sides into the microcentrifuge tubes. The samples were matrix equilibrated with the opposite matrix (50 µL of donor sample in human CVF was diluted with 50 µL PBS, while 50 µL of acceptor sample in PBS was diluted with 50 µL of human CVF). The samples were further diluted with 100 µL acetonitrile: methanol (1:1) containing IS *d*_6_-MK-2048, vortexed for 5 min at 1000 rpm, and centrifuged at 23,100× *g* for 10 min to pellet the precipitated proteins. The 100 µL supernatant was diluted with 500 µL 0.1% formic acid and used for the LC-MS/MS analysis. The percent bound of MK-2048 to human CVF proteins was determined through Equation (6).

(6) $$Bound\;\left(\%\right)=\frac{Peak\;area\;ratio\;in\;donor\;compt.-Peak\;area\;ratio\;in\;acceptor\;compt.}{Peak\;area\;ratio\;in\;donor\;compt.}\times 100\;$$

### 2.12. Statistical Analysis

The statistical analysis was performed with an unpaired Student’s *t*-test using GraphPad Prism version 10.0 (GraphPad Software, La Jolla, CA, USA). A *p*-value < 0.05 was considered statistically significant.

## 3. Results

### 3.1. Bidirectional Transport of MK-2048 Across MDCKII Cell Monolayers

**Figure 2:**
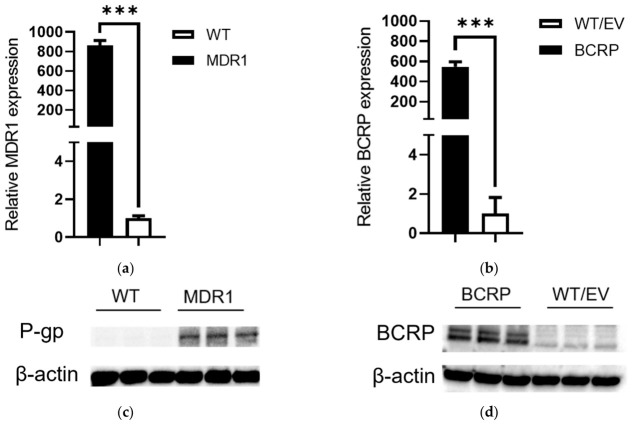
Expression of P-gp (MDR1) and BCRP at mRNA (**a**,**b**) and protein (**c**,**d**) levels in MDCKII cells overexpressing P-gp (MDR1) or BCRP and their respective control MDCKII cells (WT and WT/EV). Data are represented as mean ± SD with *n* = 6. *** *p* < 0.001.

**Figure 3:**
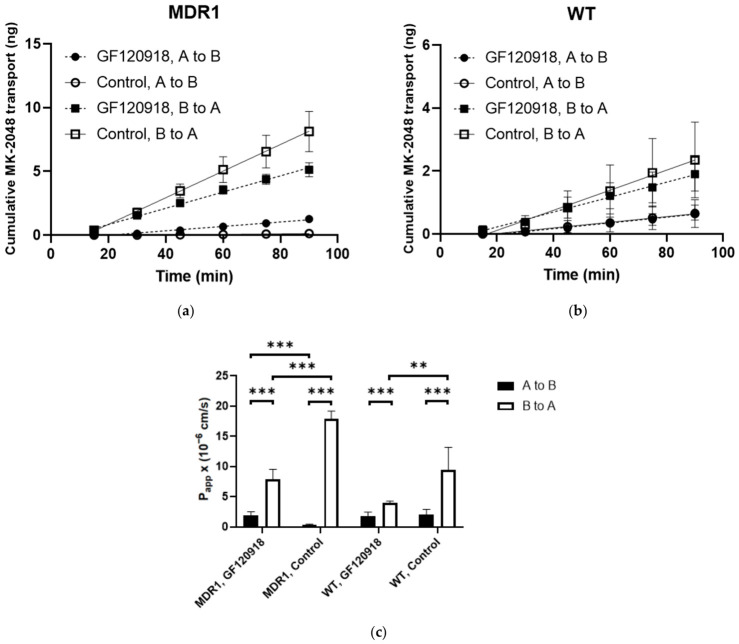
Bidirectional transport of MK-2048 across MDCKII MDR1 (**a**) and WT (**b**) cell monolayers. GF120918 is used to inhibit P-gp transporter. (**c**) P_app_ values are obtained from transport assays (*n* = 4 to 6). ** *p* < 0.01, *** *p* < 0.001.

A well-reported cellular system, an MDCKII cell line, was used to study the potential interaction between MK-2048 and P-gp/BCRP [41,42,43]. P-gp and BCRP overexpression in our MDCKII cells was confirmed as shown in Figure 2. At the mRNA level, the P-gp expression in the MDCKII MDR1 cells was 864-fold of that in the WT cells, while the BCRP expression in the MDCKII BCRP cells was 546-fold of that in the WT/EV cells. The results from the Western blot also confirmed the overexpression of P-gp and BCRP in the MDCKII MDR1 and BCRP cells at the protein level. The original images of the Western blots can be found in Supplemental Information S2. These results support the use of our MDCKII cells to study the potential interaction between MK-2048 and P-gp/BCRP. The linear transport of MK-2048 across MDCKII MDR1 and WT cell monolayers are shown in Figure 3. The calculated P_app_ values are shown in Table S4. Across the MDCKII MDR1 cell monolayer, the B-to-A flux of MK-2048 was higher than the A-to-B flux. Upon co-administration with GF120918, a known P-gp inhibitor, the B-to-A flux of MK-2048 was reduced while the A-to-B flux was increased (Figure 3a). Similarly, the B-to-A flux of MK-2048 across the MDCKII WT cell monolayer was also higher than the A-to-B flux, and this efflux was reduced by GF120918 (Figure 3b). These observations were due to the endogenous MDR1 expression in the MDCKII WT cells. As shown in Figure 3c, the P_app_ value of B-to-A transport was significantly higher than that of A-to-B transport, resulting in an ER of 57.1. GF120918 reduced the ER to 4.0, which was 7% of that of the control. The baseline ER of MK-2048 obtained with the MDCKII WT cells was 4.7, resulting in a net flux ratio of 12.1, which indicates the active efflux of MK-2048 by P-gp.

**Figure 4:**
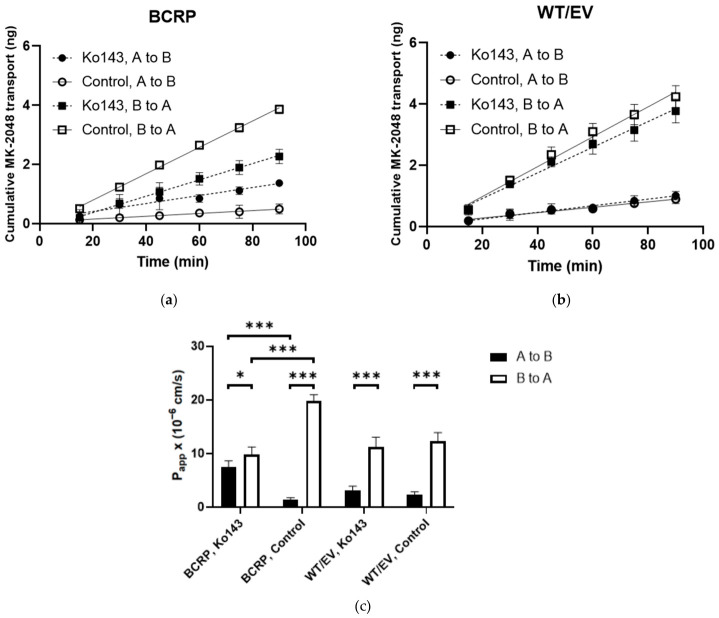
Bidirectional transport of MK-2048 across MDCKII BCRP (**a**) and WT/EV (**b**) cell monolayers. Ko143 is used to inhibit BCRP transporter. (**c**) P_app_ values are obtained from transport assays (*n* = 4 to 6). * *p* < 0.05. *** *p* < 0.001.

The linear transport of MK-2048 across the MDCKII BCRP and WT/EV cell monolayers is shown in Figure 4. The calculated P_app_ values are shown in Table S4. Across the MDCKII BCRP cell monolayer, the B-to-A flux of MK-2048 was higher than the A-to-B flux. Upon co-administration with Ko143, a known BCRP inhibitor, the B-to-A flux of MK-2048 was reduced while the A-to-B flux was increased (Figure 4a). the Across MDCKII WT/EV monolayer, due to the endogenous expression of MDR1 and the observed interaction between P-gp and MK-2048 described above, the B-to-A flux of MK-2048 was also higher than the A-to-B flux (Figure 4b). The flux was not affected by Ko143. As shown in Figure 4c, the P_app_ value of B-to-A transport was significantly higher than that of A-to-B transport, resulting in an ER of 13.9. Ko143 reduced the ER to 1.3, which was 9% of that of the control. The baseline ER of MK-2048 obtained with the MDCKII WT/EV cells was 5.3, resulting in a net flux ratio of 2.6, which indicates the active efflux of MK-2048 by BCRP.

### 3.2. Uptake of MK-2048 in MRP4 Membrane Vesicles

**Figure 5:**
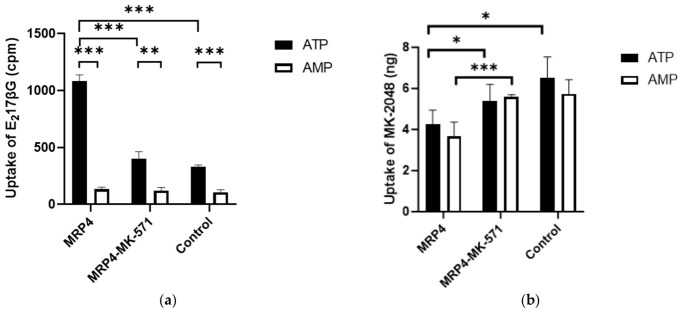
Vesicular uptake of (**a**) known MRP4 substrate, E_2_17βG, and (**b**) MK-2048 (*n* = 4 to 6). * *p* < 0.05. ** *p* < 0.01. *** *p* < 0.001.

We used an inside-out vesicular system to study the potential interaction between MK-2048 and MRP4. The vesicular uptake of MK-2048 together with the positive control, E_2_17βG, is shown in Figure 5. The uptake of E_2_17βG with ATP was significantly different from that with AMP, indicating that E_2_17βG uptake was ATP-dependent. The mean values of normalized E_2_17βG uptake were 952 cpm by the MRP4 vesicles, 279 cpm by the MRP4 vesicles co-incubated with MK-571, and 224 cpm by the control vesicles. The active uptake by the MRP4 vesicles was 4.3-fold of that by the control vesicles, and the uptake was reduced to 30% by MK-571, a known MRP4 inhibitor (Figure 5a). This validates our vesicular system for identifying MRP4 substrates. In contrast, the uptake of MK-2048 with ATP was similar to that with AMP, indicating that MK-2048 uptake was ATP-independent (Figure 5b). Therefore, the between-group difference in the MK-2048 uptake was mainly due to passive diffusion and not because of the active transport of MRP4.

### 3.3. Metabolism of MK-2048 in rCYP1A1 and rCYP1B1 Enzymes

**Figure 6:**
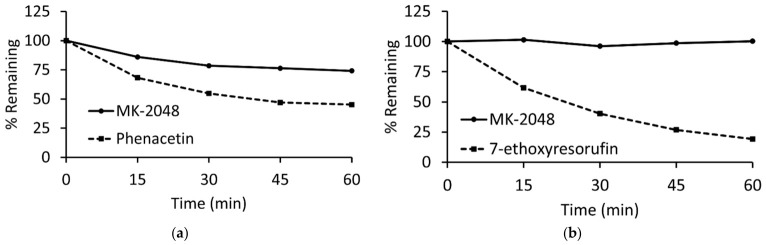
Representation of % drug remaining against time profiles in recombinant CYP 1A1 (**a**) and 1B1 (**b**) enzymes. Data are analyzed as mean of duplicate incubations.

We used recombinant enzymes to perform reaction phenotyping to study the potential interaction between MK-2048 and CYP1A1/1B1. The metabolism of MK-2048 along with the known substrates phenacetin (for rCYP1A1) and 7-ethoxyresorufin (for rCYP1B1) are shown in Figure 6. The mean % remaining of MK-2048 at 60 min in the presence of NADPH was found to be 74.11 and 100.29 in the rCYP1A1 and rCYP1B1 enzymes, respectively, which demonstrates that the metabolism of MK-2048 was higher in the rCYP1A1 enzyme than the rCYP1B1 enzyme. The mean % remaining at 60 min without NADPH (negative control) was observed as 98.33 and 95.52 in the rCYP1A1 and rCYP1B1 enzymes, respectively. According to the US FDA criteria for reaction phenotyping studies, an investigational drug is considered a substrate if the specific metabolizing enzyme contributes to ≥25% of a drug’s elimination. Based on these criteria, MK-2048 is a substrate of CYP1A1 enzyme and not a substrate of CYP1B1 enzyme. The in vitro t_1/2_ and in vitro CL_int_ of MK-2048 in the rCYP1A1 enzyme were found to be 147.99 min and 0.05 µL/min/pmol, respectively. The in vitro t_1/2_ of phenacetin and 7-ethoxyresorufin in the recombinant enzymes was 53.09 and 25.23 min, respectively.

### 3.4. Solubility and Protein Binding of MK-2048 in Human Cervicovaginal Fluid

It is well recognized that only soluble unbound drugs can reach a target site and exert their pharmacological action. Therefore, it is important to evaluate a drug’s solubility and unbound drug concentration in a bio-relevant matrix in pharmacokinetic studies. The thermodynamic solubility of MK-2048 in human CVF for 18 h was found to be 44.15 ± 5.92 µM. Protein binding of MK-2048 in human CVF was determined using an equilibrium dialysis method with the RED device inserts. The protein binding of MK-2048 in human CVF was found to be 84.75 ± 3.00%. The % recovery of MK-2048 in the protein binding study was >95.

## 4. Discussion

Efflux transporters limit their drug substrate’s penetration by pumping the drug substrates out from cells. In cervicovaginal tissue, many efflux transporters are expressed in the FRT epithelium [31]. As an integrase inhibitor that blocks viral genome insertion into the host cell DNA, MK-2048 must permeate the FRT epithelium barrier to reach the target cells to exerting its mechanism of action. Therefore, it is crucial to understand the mechanism behind MK-2048 transport across the epithelium. According to guidelines from the FDA, a substrate of efflux transporters should have a net flux ratio higher than two and a flux that will be significantly inhibited by known transporter inhibitors (the ER reduces to <50% in the presence of inhibitors) [46]. Our study confirms that MK-2048 is a substrate of both P-gp and BCRP transporters. MK-571 significantly increased MK-2048 uptake in MRP4 vesicles, both with and without the presence of ATP, suggesting passive effects that could be attributable to elevated baseline diffusion or membrane partitioning. This might have partially masked the MRP4 inhibitory effects of MK-571; nevertheless, the sensitivity of MRP4 vesicular assay was not affected, and a significant inhibitory effect of MK-571 on the MRP4 vesicular uptake of E_2_17βG, a known MRP4 substrate, was observed. The uptake of E_2_17βG in the MRP4 vesicles was significantly affected by the presence of ATP. In contrast, the uptake of MK-2048 in the MRP4 vesicles was ATP-independent. Therefore, MK-2048 is unlikely to be a substrate of MRP4. The transporter profile of MK-2048 is very similar to that of the first-generation integrase inhibitor raltegravir, which is also a P-gp and BCRP substrate but not an MRP4 substrate [34,47].

The disposition and exposure of drug substrates with low solubility or low permeability are known to be greatly affected by drug transporters. It has been reported that raltegravir accumulation in tissue can be significantly enhanced by the concurrent administration of P-gp and BCRP inhibitors, indicating the active role of P-gp/BCRP in limiting raltegravir penetration into tissue [47]. Our study reveals that MK-2048 is highly protein bound in human CVF with low solubility. Also, MK-2048 had a mean value of P_app_ of 2.0 × 10^−6^ cm/s across the MDCKII WT cell monolayer. The reported P_app_ values of many known compounds with low permeability are between 0.4 and 16 × 10^−6^ cm/s across cell monolayers [48]. Based on the solubility and permeability data, together with its structural similarity to raltegravir, the absorption and distribution of MK-2048 may also be greatly affected by drug transporters.

Considering that both P-gp and BCRP are highly expressed in the human FRT, the failure of MK-2048 in the ex vivo challenge assay in the MTN-027 trial might have been due to an insufficient MK-2048 tissue concentration because of the active efflux of MK-2048 by cervicovaginal epithelial cells [18]. Thus, higher tissue levels of MK-2048 might be needed to achieve the desired therapeutic effect. However, due to the low solubility and high protein binding of MK-2048 in human CVF, a simple dose increase in MK-2048 may not lead to sufficient MK-2048 tissue concentrations. Therefore, novel formulations that can avoid/inhibit MK-2048 efflux by cervicovaginal epithelial cells will be promising strategies to achieve sufficient MK-2048 tissue concentrations for HIV prevention. Our previous nanoparticle formulation, which encapsulated MK-2048 and prevented its binding to efflux transporters, is one such successful example demonstrating enhanced tissue penetration [36]. Currently, we are developing additional alternative formulations that can be scaled up easier and, hence, be more affordable for women in low- and middle-income areas.

Drug transporters are also responsible for many complications due to drug–drug interactions or variations in transporter expression levels. In clinical practice, combinational use of antiretroviral drugs is common in both HIV prevention and treatment [18,19,49,50]. Vicriviroc is another active ingredient in the vaginal ring delivering MK-2048 [18,19]. Vicriviroc is reported to inhibit the activity of P-gp/BCRP, and therefore could potentially enhance MK-2048 penetration into local mucosal sites [51]. In addition, many P-gp substrates, such as verapamil and cyclosporine, are also reported to be P-gp inhibitors since they compete for P-gp binding pockets [52]. Since many antiretroviral drugs are reported to be substrates of P-gp/BCRP, their concomitant use with MK-2048 could potentially alter their absorption and distribution at local mucosal sites and, thus, additional drug–drug interaction studies will be needed [29,53]. In addition, the expression of P-gp and BCRP are known to be altered under certain disease states, including inflammation and viral infection [49]. As a consequence, the tissue absorption and distribution of MK-2048 may also change under these states. Therefore, more studies need to be performed to evaluate if dose adjustment of MK-2048 is necessary under different circumstances.

CYP enzymes contribute to 70–80% of the metabolism of all clinically used drugs, including the antiretroviral drugs used to treat HIV [54]. Dolutegravir, an integrase inhibitor, showed major metabolism by CYP 1A1 and 1B1 enzymes [38]. In this work, we found that MK-2048 has significant metabolism by CYP1A1 compared to the CYP1B1 enzyme. The CYP 1A1 and 1B1 enzymes have higher expressions in cervicovaginal tissues [30,37]. These enzymes can be induced by cigarette smoking, and the clearance of dolutegravir was higher in smokers than non-smokers [38], suggesting that smokers may require higher dosing of MK-2048 to achieve therapeutic efficacy compared to non-smokers.

Characterization of MK-2048 has a broader utility for vaginal dosage form development. Our study identifies MK-2048 as a sensitive probe substrate for P-gp, BCRP, and CYP1A1, suggesting its broad applicability to studying the drug–drug interactions mediated by these pathways. In addition, our published work on nanoparticles [36] and unpublished preliminary data suggest efflux of MK-2048 can be effectively mitigated through targeted formulation strategies. When combined with its established clinical safety profile and demonstrated suppression of HIV-1 replication in cellular and tissue models, these strategies may enable the development of formulations with enhanced tissue permeability and mucosal absorption, thereby potentially revitalizing MK-2048 as a safe and effective anti-HIV agent.

## 5. Conclusions

In summary, using MDCKII cell lines, our study reveals that MK-2048 is a substrate of P-gp/BCRP and the efflux of MK-2048 by P-gp and BCRP can be inhibited by known P-gp/BCRP inhibitors. MK-2048 is also found to be a substrate of the CYP1A1 enzyme. This is the first comprehensive report discussing the in vitro pharmacokinetic parameters of MK-2048, including its transport, metabolism, solubility, and protein binding. Understanding these parameters is essential for predicting MK-2048 disposition in the human FRT and developing effective vaginal dosage forms containing MK-2048. We demonstrate the role of both transporters and metabolic enzymes in limiting the distribution of MK-2048 in the human FRT. These findings highlight the importance of efflux transporters and metabolic enzymes in the accumulation of MK-2048 in cervicovaginal tissues.

## Data Availability

A blank informed consent form used in the CVF collection protocol and Supplemental Data can be found in Supplemental Information S1 and S2, respectively. Additional data supporting the reported results will be made available from the corresponding author upon request (Lisa C. Rohan).

## Supplementary Materials

The following supporting information can be downloaded at: https://www.mdpi.com/article/10.3390/v18050561/s1, Information S1: A blank informed consent form for the IRB approved clinical protocol for collection of cervicovaginal fluid (PRO10080337; approval date: 25 June 2019); Information S2: Figure S1: Representative calibration curve of MK-2048; Figure S2: Original Western Blot images for P-gp; Figure S3: Original Western Blot images for BCRP; Table S1: Linearity of the MK-2048 LC-MS/MS method across three different days; Table S2: Accuracy of the MK-2048 LC-MS/MS method across three different days; Table S3: Precision of the MK-2048 LC-MS/MS method across three different days; Table S4: Mean (SD) apparent permeability (P_app_) and efflux ratios in P-gp/BCRP permeability assays.
