# Supplementary material for: In Vitro Pharmacokinetic Properties of MK-2048, a Potent Drug Candidate for HIV Prevention
Source: Viruses. 2026 May 15;18(5):561. doi: 10.3390/v18050561 (PMC13211645; doi:10.3390/v18050561)
Supplement: Supplementary file 1 [file viruses-18-00561-s001.zip › MK2048 transport and metabolism_Supplemental Information S1.pdf]

Magee-Womens Hospital
of University of Pittsburgh Medical Center
300 Halket Street   Pittsburgh, PA 15213-3180

CONSENT TO ACT AS A PARTICIPANT IN A RESEARCH STUDY

Title: Collection of Biological Specimens for Development and Validation of Laboratory Procedures to Support Future Clinical Research Studies
Short Title: Specimen Collection Study

Consent Version #: 10.0 20Jun2019

**Principal Investigator:**	Katherine Bunge, MD<br>Magee-Womens Hospital of UPMC<br>Department of OB/GYN/RS<br>300 Halket Street, Pittsburgh, PA 15213<br>Phone: 412-641-4242

The main purpose of this research study is to collect samples from healthy women so that research investigators can use the samples in the laboratory (e.g. to validate processes or to develop research laboratory tests) in preparation for future studies evaluating STDs (Sexually Transmitted Diseases), including Human Immunodeficiency Virus (HIV).

This study only involves the collection of samples. No study medications are being tested directly on women who agree to participate in this study. This study is being funded by the National Institutes of Health and through Departmental Funding.

Up to 500 non-pregnant women between the ages of 18-45 or postmenopausal women over 50 years of age who agree to have HIV testing (or who have a negative HIV test result from the past 6 months) will be invited to participate. Study visits will occur at Magee-Womens Hospital of UPMC, Clinical Translational Research Center (CTRC), Suite 5500 or ancillary research space. Each visit can take up to one hour depending on the samples collected at that visit. Each participant can be in the study for up to 6 months from the date of her HIV negative test result, and have up to six study visits, depending on the laboratory's need for samples.   At the end of the six months, women may be asked to re-enroll in the study by signing another consent form and having another HIV test.

If you agree to participate, the following will happen today:

- If you have written confirmation of HIV negative test results that have been performed in the past six months, you will be asked for a copy of the test result. If you do not have a copy or have not had recent (past 6 months) HIV testing, you will undergo HIV testing as part of this study, as detailed below.

  HIV testing
  If you agree to participate and you qualify for the study, you will undergo pre-test HIV counseling followed by rapid HIV testing, either through blood or by mouth (saliva) testing using Orasure. If the test is done through your blood, approximately one teaspoon (4mL) of blood will be drawn from a vein in your arm or hand. If the test is done by an oral test, you will be asked to use a special pad to collect a sample from your gums/inner cheek.

  After it is collected, your sample (blood or saliva) will be tested for the antibody to HIV. An antibody is a substance that blood cells make to fight infection. Exposure (contact) to the HIV virus produces antibodies. A positive HIV test means that the sample tested positive for

University Of Pittsburgh
Institutional Review Board
Approval Date: 6/25/2019
Renewal Date: 4/23/2020
IRB #: PRO10080337

antibodies to HIV. Repeat testing would be necessary to confirm (prove) this finding. If the sample is positive for antibodies to HIV, it means that the person has been exposed to HIV and is a carrier of HIV. It also means that the virus can be passed to others by intimate sexual contact, by sharing needles and through donating blood and organs. A negative HIV test means that at this time, no antibody to HIV was found in the blood sample based on the result of the initial screening test.

There can be individuals who have HIV test results that are called "false positive," (for some reason the test shows that HIV antibodies are present in the blood when they are not). There can also be false negative results which can have two possible meanings, 1) the person has been infected with HIV, but that person's body has not yet made antibodies to the virus, or 2) HIV antibody is present in the person's blood, but for some reason the test failed to detect it.

The HIV tests used for this study are rapid tests and the results will be available in about twenty minutes from the time the sample is taken. We will give you your results in person and the results will be available today. If your test is positive for HIV antibody, you may be asked to give 2 tablespoons of blood for a repeat HIV antibody test or you may be referred for confirmatory testing (e.g. Allegheny County Health Department, Sexually Transmitted Diseases Clinic). You will also be counseled as to the risks for transmitting HIV to others, risks for developing AIDS and the available treatments for HIV infection. This study does not provide evaluation or treatment for HIV, but study staff will refer you to available sources of medical care, counseling, and other services you may need if your test is positive. Study staff also will be available to talk with other doctors that you see for your medical care and share your test results (with your permission). You will not be able to participate in this study as your HIV test result must be negative in order to participate in this study.

- You will be asked a brief questionnaire, including questions such as age, race, general medical history and sexual history questions, medication and birth control use. You may refuse to answer any question(s) you like.
- You will have a urine pregnancy test done at this visit and at any subsequent study visits over the next six months when samples are collected. Your pregnancy test must be negative in order to participate in the study. If you become pregnant while you are participating in this study, the researchers will no longer be able to collect samples from you.
- You may have STD testing (gonorrhea and chlamydia) done at this visit and at any subsequent study visits over the next six months when samples are collected.   You will be notified and counseled if your test result is positive. You will be responsible for the cost of treatment associated with a positive test result. Positive STD and HIV test results are reported to the Allegheny County Health Department, according to the Commonwealth of Pennsylvania reporting requirements. Participants who are positive may be contacted by the health department to ask questions related to sexual partners.
- You may have vaginal swabs collected to verify if you have a vaginal infection (i.e. an investigator may look at the sample under the microscope to assess the bacteria in the vagina).
- Detailed contact information (home/cell phone numbers, address, email, alternate contacts) will be collected from you; staff will remind you about appointments and if necessary will notify you of test results.
- You must consent to at least one of the research procedures listed below to participate in this study. The samples will be collected in a private examination room by an individual trained in performing the procedure.

University Of Pittsburgh
Institutional Review Board
Approval Date: 6/25/2019
Renewal Date: 4/23/2020
IRB #: PRO10080337

- After you have a chance to read and understand each procedure, you will be asked to agree or not agree to the procedures by initialing each box on the last page (Voluntary Consent) of this consent.

OPTIONAL PROCEDURES:

Urine Collection →a urine sample will be collected (as much as you are able to provide at the time of the visit)

Blood Collection → Up to 6 teaspoons of blood (30mL) will be drawn from a vein in your arm or hand.

Throat Samples → Up to 3 throat sampleswill be collected per clinic visit.

Vaginal Samples → Up to 8 vaginal sampleswill be collected per clinic visit.

Cervical Samples → Up to 6 cervical sampleswill be collected per clinic visit.

Vaginal/Cervical Fluid Collection → Fluid will be collected using a menstrual cup/discdevice (similar looking to a diaphragm). The menstrual cup/disc is an FDA approved device that is used for women outside the study for menstrual protection, or to collect blood during a period. You will be instructed on how to insert the menstrual cup or alternately a clinician may insert it for you. You may be asked to leave the cup inserted up to 8 hours. The length of time will be determined by the needs of the laboratory at that timeAfter this time period, a clinician will remove the cup and collect any fluid that has collected in the cup.

Cervicovaginal Lavage → Approximately 2 teaspoons (10mL) of sterile water will be inserted into the vagina while a speculum is in place. The water will be flushed in the vagina for approximately one minute and then will be collected from the vagina.

Cervical Aspirate → A plastic straw-like instrument will be a used to draw the mucus from the cervix.

Vaginal Biopsy → Up to two vaginal biopsies (approximately 3mm by 5mm each) will be taken from the upper to mid-vagina using a biopsy instrument. Some of the samples may be collected after applying an anesthetic (like Novocain) to the surface to numb the tissue. In some instances, the samples may need to be taken without the use of an anesthetic. You can choose how and if you want these samples collected. The biopsies take a few seconds to do and will be done by a physician experienced at performing them.

Cervical Biopsy → Up to two cervical biopsies (approximately 3mm by 5mm each) will be taken from the cervix using a biopsy instrument. Some of the samples may be collected after applying an anesthetic (like Novocain) to the surface to numb the tissue. In some instances, the samples may need to be taken without the use of an anesthetic. You can choose how and if you want these samples collected. The biopsies take a few seconds to do and will be done by a physician experienced in performing them.

Rectal Swabs → Up to 3 rectal swabs, similar to Q-tips, will be collected.

- If you choose to have genital samples collected, a pelvic examination will be performed prior to the collection of the samples which will include insertion of a speculum (an instrument that is inserted into the vagina to visualize the vagina and cervix). Depending on the laboratory needs at the time of specimen collection, if you have signs of an infection (e.g. abnormal vaginal discharge) or symptoms of a vaginal infection, you may not be able to have your samples collected that day. You may be referred to a clinic to further evaluation and possible treatment.
- **The samples that the laboratory needs may vary from month to month** (e.g one month blood may be needed; another month blood and vaginal tissue may be needed). If you choose more than one sample, one or more of the procedures may be collected at a single visit (e.g. blood draw only or blood draw plus vaginal biopsies), depending on the need for samples. On the other hand, if you are willing to have all the procedures collected but only blood samples are needed during the six months you are in the study, then only blood will be collected.
- If you consent to have a procedure done, for example a vaginal biopsy, but following the procedure decide that the biopsy was too uncomfortable and you do not wish to have it repeated for the study, this decision will not impact your ability to have other samples collected in the future which you agreed to (e.g. a blood draw).

Study information (collected on questionnaires) and study specimens collected as part of this study will be shared with other investigators. The information and specimens will only be made available to other investigators by study number and will not include any personal identifying information.

All of the samples for this study will be taken to Magee Womens Research Institute. The majority of the samples will be processed in laboratories at the Research Institute. However, there may be study samples that will be sent from the Research Institute to outside laboratories for processing and analysis. It is important for you to remember that these samples will only be given to these laboratories with your study number, not personal identifiers, in order to protect your identity.

The results from the testing for research purposes will not be given to you and will not become part of your medical record as these tests would have no impact on your clinical care or clinical decision making.

Study specimens may be stored for an indefinite period of time so that research investigators can use the samples in the laboratory (e.g. to validate processes or to develop research laboratory tests) in preparation for future studies evaluating STDs (Sexually Transmitted Diseases), including Human Immunodeficiency Virus (HIV).

As with any research study, there may be adverse events or side effects that are currently unknown and it is possible that certain of these unknown risks could be permanent, serious, or life-threatening.

<u>Answering Research Questionnaires</u> → May be uncomfortable or cause embarrassment.

<u>Pregnancy Testing</u> → You may become worried or anxious waiting for test results. Getting an unexpected positive pregnancy test result can cause feelings of denial, depression and confusion.

<u>STD and HIV Testing</u> → You may become worried or anxious waiting for test results. Testing positive for an STD or HIV may commonly cause feelings of sadness, depression or denial and may infrequently case feelings of isolation. Being told a person has HIV has been associated with depression, suicidal ideation and denial as well as feeling isolated from society.

University Of Pittsburgh
Institutional Review Board
Approval Date: 6/25/2019
Renewal Date: 4/23/2020
IRB #: PRO10080337

Blood Draw → May commonly cause bruising, bleeding, pain or discomfort at the site of the blood draw. Infrequently may cause fainting and rarely a local infection.

Throat Swabs → Collecting throat swabs may be uncomfortable or cause you to gag and/or vomit.

Vaginal and Cervical Samples → Collecting vaginal and/or cervical samples may cause minimal discomfort and/or minimal spotting but is very well tolerated. The discomfort is more likely due to inserting the speculum (which is the instrument used to visualize the vagina and the cervix) for the pelvic examination.
Cervicovaginal Lavage (CVL)/Cervical Aspirate → May be associated with minimal discomfort most likely due to the speculum in place during the procedure. Otherwise CVL/Cervical Aspirate is very well tolerated.

Vaginal/Cervical Fluid collection with the menstrual cup/disc→ May be uncomfortable to insert and/or remove.

Vaginal and Cervical Biopsy → May cause mild to moderate discomfort or pain; which may be worse without the use of analgesics (e.g. ibuprofen) or topical anesthetics (Novocain). May also cause bleeding and much more unlikely an infection or scar formation. If you are given ibuprofen, it may cause an upset stomach. Alternately, if acetaminophen is used, it is unlikely to cause any side effects, especially from a single dose. Even though unlikely, nausea, stomach pain or rash could occur.

Any cervical procedure listed above (i.e. cervical biopsy, cervical aspirate) has the small, but rare risk of dislodging an Intrauterine Device (IUD).

Rectal Swabs → collecting rectal swabs may cause discomfort or minimal bleeding.

Participating in research → May be inconvenient or cause a breach of confidentiality meaning that others may know you are participating in this study. Research staff will do everything possible to protect your privacy.

You may not directly benefit from participating in this research study. If a study doctor finds something abnormal, you will be referred to your primary care provider or another healthcare provider for further evaluation or treatment. The results from this study may add to the current knowledge available to investigators and may also be used to develop new methods to prevent STDs, including HIV.

You will promptly be notified if any new information develops during the course of this study which may cause you to change your mind about continuing to participate.

All research-only visits, procedures and associated laboratory tests associated with this study will be paid for by this study. If you get a bill or believe your health insurance has been billed for something that is part of this study, notify the study staff or UPMC Patient Billing Services.

You will be compensated each time you provide study samples, as they are needed, during your six months of study participation.

Urine sample → $10
Blood sample → $20

University Of Pittsburgh
Institutional Review Board
Approval Date: 6/25/2019
Renewal Date: 4/23/2020
IRB #: PRO10080337

Throat swabs → $10 for every 3 samples collected
Vaginal Swabs → $10 for every 4 samples collected
Cervical Swabs and/or Brushes → $10 for every 3 samples collected
Vaginal/Cervical Fluid Collection → $30
Cervicovaginal Lavage/Cervical Aspirate → $20
Vaginal Biopsy → $40 per biopsy
Cervical Biopsy → $40 per biopsy
Rectal swabs → $10 for every 3 swabs collected

Total compensation among participants will vary depending on: 1). the samples a participant chooses to have collected **and** 2). the investigator's need to collect the samples during the 6 months of participation.

Since you are being compensated for your participation in this study, your name, address, and social security number will be released to the Accounting Office. If the total reimbursement for your participation in research is greater than $600 in a year, this will be reported to the Internal Revenue Service (IRS) as income.

If investigators are able to develop new products from the research use of your samples, there are currently no plans to share with you any money or other rewards that may result from the development of new products.

Your doctor may also be involved as an investigator in this research study, but **you are not under any obligation to participate in any research study offered by your doctor**. Before agreeing to participate in this research study, or at any time thereafter, you may wish to discuss participation in this study with another health professional, to obtain a 'second opinion' about study participation.

If you believe that the research procedures have resulted in an injury to you, immediately contact Dr. Hillier or the study team (412-641-4242). Emergency medical treatment for injuries solely and directly related to your participation in this research study will be provided to you by the hospitals of UPMC. Your insurance provider may be billed for the costs of this emergency treatment, but none of those costs will be charged directly to you. If your research-related injury requires medical care beyond this emergency treatment, you will be responsible for the costs of this follow-up care. At this time, there is no plan for any additional financial compensation.

Identifiable information (e.g. name, date of birth, address) will be collected from you as part of your participation in this study for purposes of ensuring that you qualify (date of birth), for scheduling purposes and to provide compensation to you (payments loaded onto cash cards through the Vincent system). This information will be made available to members of the research team, for an indefinite period of time and may be shared with other groups, possibly including the University of Pittsburgh Research Conduct and Compliance Office, and the National Institutes of Health, but only for the purpose of monitoring the study.

Although we will do everything in our power to protect your privacy and the confidentiality of your research records, just as with the use of your medical information for health care purposes, we cannot guarantee the privacy of your research records, including information that we obtained from your medical records.

Your participation in this research study is completely voluntary. Whether or not you participate in this research study will have no effect on your current or future relationship with the University of Pittsburgh, UPMC, or its affiliated health care providers or health care insurance providers. If you decide you no longer wish to continue to participate after you have signed the consent form, you should contact Dr. Hillier or his research staff (412-641-4242). If your specimens are still available you may request that they be discarded according to the routine practice of the University of Pittsburgh Medical Center. Your decision to withdraw from this study will have no effect on your current or future relationship with the University of Pittsburgh or with UPMC or its affiliate health care and insurance operations. If the investigators feel that you cannot complete the study requirements safely, they may withdraw you from the study. You may also be withdrawn if you are pregnant.

****

Please indicate below, by initialing the appropriate box(es), which procedures you are willing to undergo for this research study. You must consent to at least one of the procedures to participate in this study; you are under no obligation to agree to any more than one. Please understand that the need for specimens during your six months of participation may require that you come in for separate visits.

Initial if you AGREE	Initial if you DO NOT AGREE	Optional Research Study Procedures
		Urine sample
		Blood draw
		Throat samples
		Vaginal samples
		Cervical samples
		Cervicovaginal Lavage (CVL)
		Cervical Aspirate
		Vaginal/Cervical Fluid collection with menstrual cup/disc
		Vaginal Biopsy –with anesthetic
		Vaginal Biopsy – without anesthetic
		Cervical Biopsy – with anesthetic
		Cervical Biopsy – without anesthetic
		Rectal swabs

VOLUNTARY CONSENT

The above information has been explained to me and all of my current questions have been answered. I understand that I am encouraged to ask questions, voice concerns or complaints about any aspect of this research study during the course of this study, and that such future questions, concerns or complaints will be answered by a qualified member of the research team or by the principal investigator listed on the first page. I understand that I may always request that my questions, concerns or complaints be addressed by the principal investigator. At any time I may also contact the Human Subjects Protection Advocate of the IRB Office, University of Pittsburgh (1-866-212-2668) to discuss problems, concerns and questions; obtain information; offer input; or discuss situations in the event that the research team is unavailable. **By signing this form I agree to participate in this research study and allow the use and disclosure of my medical record information for the purposes described above.** A copy of this consent form will be given to me.

____
Printed Name of Participant

____ _____________ _____________
Participant's Signature Date Time (am/pm)

CERTIFICATION OF INFORMED CONSENT:

I certify that I have explained the nature and purpose of this research to the above individual and I have discussed the potential benefits and possible risks of study participation. Any questions the individual has about this study have been answered, and we will always be available to address future questions as they arise. I further certify that no research component of this protocol was begun until after this consent form was signed.

____ ____________________
Printed Name of Person Obtaining Consent Role in Research Study

______________________________________ ____________________
Signature of Person Obtaining Consent Date

University Of Pittsburgh
Institutional Review Board
Approval Date: 6/25/2019
Renewal Date: 4/23/2020
IRB #: PRO10080337
