# Supplementary material for: In Vitro Pharmacokinetic Properties of MK-2048, a Potent Drug Candidate for HIV Prevention
Source: Viruses. 2026 May 15;18(5):561. doi: 10.3390/v18050561 (PMC13211645; doi:10.3390/v18050561)
Supplement: Supplementary file 1 [file viruses-18-00561-s001.zip › MK2048 transport and metabolism_Supplemental Information S2_Revised 5.5.26.pdf]

In vitro Pharmacokinetic Properties of MK-2048, a Potent Drug Candidate for HIV Prevention

Ruohui Zheng [1,2], Guru Raghavendra Valicherla [1,2], Phillip Graebing [1,2], Junmei Zhang [2,3], Sharon L Hillier [2,3], and Lisa Cencia Rohan [1,2,3,*]

1. Department of Pharmaceutical Sciences, School of Pharmacy, University of Pittsburgh, Pittsburgh, Pennsylvania, 15213, USA; ruz33@pitt.edu (R.Z.); pwg12@pitt.edu (P.G.); junmei.zhang@pitt.edu (J.Z.)
2. Magee-Womens Research Institute, Pittsburgh, Pennsylvania, 15213, USA; gururaghava810@gmail.com (G.R.V.); hillsl@mwri.magee.edu (S.H.)
3. Department of Obstetrics, Gynecology, and Reproductive Sciences, School of Medicine, University of Pittsburgh, Pittsburgh, Pennsylvania, 15213, USA;

* Correspondence: rohanlc@upmc.edu; Tel.: +1-412-641-6108

Representative calibration curves, linear range, accuracy, and precision data for the MK-2048 LC-MS/MS method are shown in Figure S1 and Tables S1-S3.

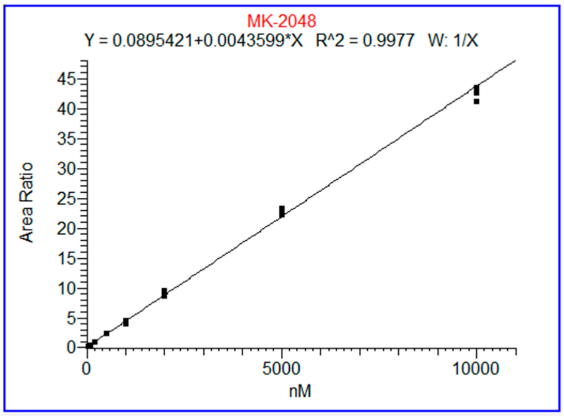

Figure S1. Representative calibration curve of MK-2048. The eight concentrations of the calibration standards were 50, 100, 200, 500, 1000, 2000, 5000, and 10000 nM.

Table S1. Linearity of the MK-2048 LC-MS/MS method across three different days.

	R^2	Slope	Intercept
07/30/2019	0.9949	0.0049905	0.0087516
08/01/2019	0.9977	0.0043599	0.0895421
08/02/2019	0.9961	0.0049302	0.0346209

Table S2. Accuracy of the MK-2048 LC-MS/MS method across three different days.

	Accuracy (%)		
	300 nM	1500 nM	7500 nM
07/30/2019	108.38±2.88	103.95±6.00	98.55±4.02
08/01/2019	104.11±2.52	106.97±5.16	106.37±4.44
08/02/2019	105.92±5.75	95.84±4.87	97.20±6.90

Table S3. Precision of the MK-2048 LC-MS/MS method across three different days.

	RSD (%)		
	300 nM	1500 nM	7500 nM
07/30/2019	2.66	5.77	4.08
08/01/2019	2.42	4.83	4.18
08/02/2019	5.75	5.08	7.10

The original Western Blot images of Figures 2c and 2d are shown in Figures S2 and S3. Please note that we used the ECL method to visualize the bound antibodies, so the protein bands had to be captured separately from the ladder with a different setting.

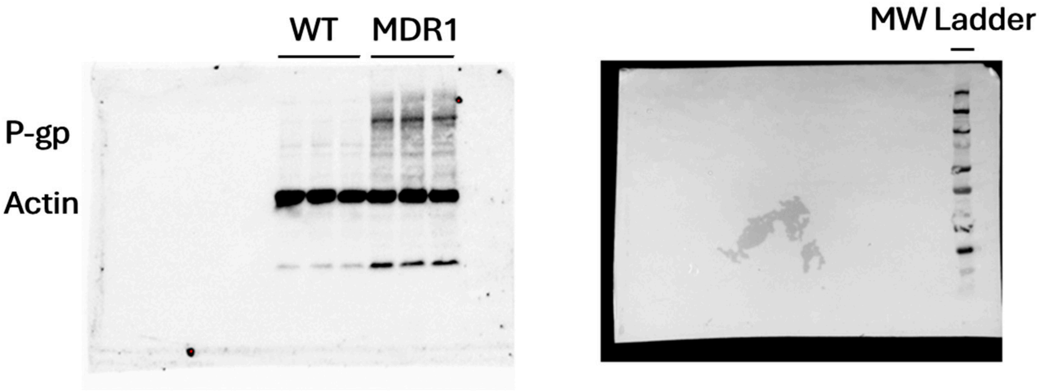

Figure S2. Original Western Blot images for P-gp

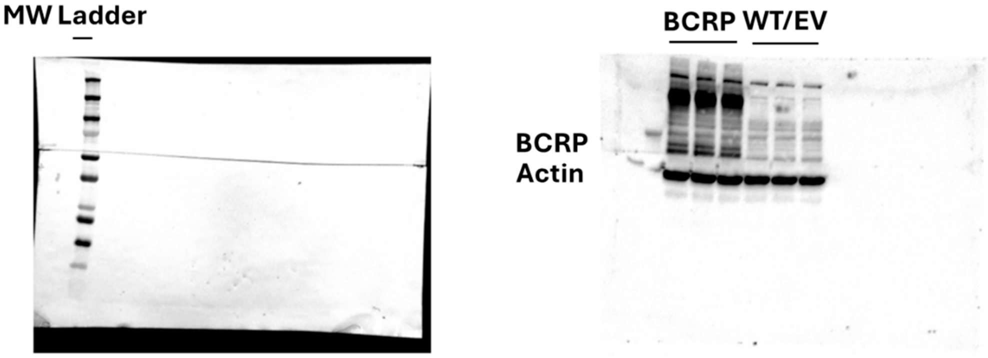

Figure S3. Original Western Blot images for BCRP

The apparent permeability (P_{app}) values used to generate Figures 3c and 4c and the calculated efflux ratios are listed in Table S4.

Table S4. Mean (SD) apparent permeability (P_{app}) and efflux ratios in P-gp/BCRP permeability assays

Transporter	Cell Line	Group	Direction	P_{app} x 10^6 (cm/s)	Efflux Ratio
P-gp	MDCKII MDR1	Control	A to B	0.313 (0.168)	57.1
			B to A	17.883 (1.317)	
		GF120918	A to B	1.970 (0.559)	4.0
			B to A	7.892 (1.655)	
	MDCKII WT	Control	A to B	2.014 (0.891)	4.7
			B to A	9.418 (3.749)	
		GF120918	A to B	1.853 (0.605)	2.2
			B to A	4.041 (0.242)	
BCRP	MDCKII BCRP	Control	A to B	1.428 (0.363)	13.9
			B to A	19.858 (1.129)	
		Ko143	A to B	7.554 (1.094)	1.3
			B to A	9.860 (1.388)	
	MDCKII WT/EV	Control	A to B	2.314 (0.557)	5.3
			B to A	12.360 (1.561)	
		Ko143	A to B	3.056 (0.857)	3.7
			B to A	11.241 (1.804)	
